# Inflammatory biomarkers as predictors of immune activation to different irradiated sites and short-term efficacy in advanced squamous cell esophageal carcinoma received radioimmunotherapy

**DOI:** 10.3389/fonc.2023.1117648

**Published:** 2023-05-10

**Authors:** Mengying Li, Guoxin Cai, Zhenhua Gao, Xue Meng, Xiao Han

**Affiliations:** ^1^Department of Radiation Oncology, Shandong Cancer Hospital and Institute, Shandong First Medical University and Shandong Academy of Medical Sciences, Jinan, Shandong, China; ^2^Department of Radiation Oncology, School of Medicine, Shandong University, Jinan, China

**Keywords:** squamous cell esophageal carcinoma, inflammatory biomarkers, immune activation, irradiated sites, short-term efficacy

## Abstract

**Purpose:**

The present study aimed to compare immune activation among different irradiated sites and identify potential short-term efficacy prognostic factors in patients with advanced squamous cell esophageal carcinoma (ESCC) who received radiotherapy (RT) and immunotherapy.

**Patients and methods:**

We recorded the clinical characteristics, blood cell counts, and derived blood index ratios, including neutrophil-to-lymphocyte ratio (NLR), lymphocyte-to-monocyte ratio (LMR), platelet-to-lymphocyte ratio (PLR), and systemic immune-inflammation index (SII), at three time points (before, during, and after RT) in 121 patients with advanced ESCC who had received RT and immunotherapy. Chi-square test and univariate and multivariate logistic regression analyses were used to calculate the relationships among inflammatory biomarkers (IBs), irradiated sites, and short-term efficacy.

**Results:**

Delta-IBs were calculated as (medio-IBs - pre-IBs) ÷ pre-IBs. The medians of delta-LMR, and delta-ALC were the highest, whereas the median of delta-SII was the lowest in patients with brain radiation. Treatment responses were observed within 3 months after RT or until the beginning of the next line therapy, and the disease control rate (DCR) was 75.2%. The areas under the receiver operating characteristic curve (AUCs) for delta-NLR and delta-SII were 0.723 (p = 0.001) and 0.725 (p < 0.001), respectively. Multivariate logistic regression analysis showed that the treatment lines of immunotherapy (odds ratio [OR], 4.852; 95% confidence interval [CI], 1.595-14.759; p = 0.005) and delta-SII (OR, 5.252; 95% CI, 1.048-26.320; p = 0.044) were independent indicators of short-term efficacy.

**Conclusion:**

In this study, we found that RT to the brain had a stronger immune activation effect than RT to extracranial organs. We also found that earlier-line immunotherapy plus RT and a decrease in SII during RT may generate better short-term efficacy in advanced ESCC.

## 1 Introduction

Squamous cell esophageal carcinoma (ESCC) is the most common histological type of esophageal cancer (EC) in China (1, 2). Most patients have advanced or metastatic disease when diagnosed, with a 5-year survival rate of less than 20%. Immune checkpoint inhibitors (ICIs), mainly targeting programmed cell death receptor-1 (PD-1) and its ligand (PD-L1), have greatly improved outcomes and have been added to the current standard of care, which includes platinum-based chemotherapy. However, the current study demonstrated that the survival benefit from ICIs alone is limited, as patients frequently develop immune resistance, regardless of whether the tumor is immunogenic or whether the microenvironment is immune-suppressive (3). Thus, combination strategies for advanced ESCC patients receiving immunotherapy are required to overcome immune resistance and achieve optimal therapeutic benefits.

Radiotherapy (RT), another pillar of advanced EC treatment, can activate the innate and adaptive immune responses by enhancing the presentation of tumor antigens and increasing T lymphocyte infiltration to potentiate the effects of immunotherapy, which involves a variety of inflammatory cells, cytokines, and chemokines in the tumor microenvironment (4–7). Zhang and colleagues found that in locally advanced ESCC, RT plus anti–PD-1 antibody as first-line therapy is safe and feasible (8). Studies have shown that systemic inflammation is a hallmark of the development and progression of malignant tumors, which usually occurs when the balance between the inflammatory cells (neutrophils and monocytes) and tumor-specific lymphocytes becomes disrupted (9, 10). RT activates pro-inflammatory factors including interferons and chemokines that attract activated T cells into tumors (11). When the anti-cancer therapy triggered by RT works, the body improves the immune status by increasing the lymphocyte count and decreasing monocytes, which leads to increased lymphocyte count and lymphocyte-to-monocyte ratio (LMR) (12). Moreover, a number of inflammatory biomarkers (IBs) and their derived ratios have been investigated as prognostic indicators in various cancers. The systemic immune inflammation index (SII), an integrated indicator based on peripheral lymphocyte, neutrophil, and platelet counts, is a strong prognostic indicator for patients with several tumor types (13). Recent studies have revealed that higher neutrophil-to-lymphocyte ratio (NLR) and platelet-to-lymphocyte ratio (PLR) are associated with poorer outcomes in ESCC (14, 15). Lymphocytes are important in promoting antitumor immunity, and a higher lymphocyte-to-monocyte ratio (LMR) generally indicates better survival and response to immunotherapy (15–17). The mentioned IBs and their derived ratios change when patients undergo RT and immunotherapy, as a result of differences in radiosensitivity among different immune cell types (18).

However, local relapses often occur following RT, suggesting RT-induced responses are inadequate to maintain antitumor immunity (19). Many preclinical studies (6, 19) have validated that locoregional tumor control increases when radiotherapy is combined with checkpoint blockade immunotherapy. Clinical studies in colorectal cancer and non-small cell lung cancer (NSCLC) also showed that RT synergized with ICIs and improved the therapeutic effect (20, 21). The increased infiltration of CD8 effector cells and increased ratio of CD8 effector cells to regulatory T cells may explain this immune-based mechanism for combinatorial efficacy (22). In addition, previous studies have reported that, as a result of spatial intratumor heterogeneity and temporal heterogeneity in ESCC, the bulk tumor might include a diverse collection of cells harboring distinct molecular signatures and cancer-related signaling pathways with differential levels of sensitivity to treatment (23–26). Inherent differences exist in the immune microenvironment of different metastatic sites, from the relatively immune-privileged brain protected by the blood-brain barrier to the lung and liver, which are constantly exposed to antigens and have a relatively immunotolerant microenvironment. Studies have also shown that stereotactic ablative RT (SAR) induces systemic immunologic changes that are dependent on the irradiated site (27). Consequently, we speculated that the synergistic effect of RT and immunotherapy on different metastases could produce different immune system changes; however, few studies have explored how to optimize RT with immunotherapy for advanced ESCC with multiple metastases to achieve the optimal combined response.

In this study, we collected the mentioned IBs to examine the immune activation effect among different irradiated sites during radioimmunotherapy and explore the potential factors related to short-term efficacy in advanced ESCC patients who received RT as well as immunotherapy.

## 2 Patients and methods

### 2.1 Study design and patients

This retrospective study reviewed data of patients who had received radiotherapy (RT) for advanced ESCC with immunotherapy at Shandong Cancer Hospital and Institute between July 2019 and December 2021. We enrolled in the study 121 patients who had received RT for primary or metastatic solid tumors after or concurrent with immunotherapy combined with chemotherapy. The inclusion criteria were as follows: (a) histologically confirmed ESCC from available biopsy specimens; (b) Karnofsky score ≥70; and (c) absence of any other primary tumor or chronic inflammatory disease. Patients with early stage or non-ESCC were excluded from the study. All patients were staged according to the eighth edition of the American Joint Committee on Cancer (AJCC) tumor, node, metastasis (TNM) staging for EC. The study was approved by the institutional review board of Shandong Cancer Hospital and Institute. The need for written informed consent was waived due to the retrospective nature of the study.

### 2.2 Treatment characteristics

All patients were treated with RT and immunotherapy, either simultaneously, or sequentially. We defined the group of patients for whom the initiation of immunotherapy preceded the first day of RT as the immunotherapy-prior-to-radiotherapy group. Concurrent radio-immunotherapy was identified as immunotherapy initiated during the interval from the first day to the 7 days after completion of RT. In this study, RT was delivered using tomographic radiation therapy or intensity-modulated radiation therapy. Patients underwent 10 to 34 fractions of conventional fractionated RT (CFRT) at 1.8-4.0 Gy per fraction, 3 to 10 fractions of stereotactic body RT (SBRT) at 5.0-12.5 Gy per fraction, or 30 to 50 fractions of hypofractionated RT (HFRT) at 1.2-1.3 Gy per fraction for the primary or metastatic site (including the drainage area or non-area lymph node with or without esophagus mass, bone, brain, liver, lung, or other organs). For systemic treatment, all patients were administered anti-PD-1 agents until disease progression or unacceptable toxicity was observed.

### 2.3 Data collection

Clinicopathological characteristics including age, sex, Karnofsky score, smoking and drinking status, TNM stage, treatment mode, immunotherapy, and RT details were extracted from the patients’ medical records. The laboratory data collected included absolute white blood cell count (WBC), absolute neutrophil count (ANC), absolute lymphocyte count (ALC), absolute monocyte count (AMC), absolute platelet count (APC), absolute eosinophil count (AEC) and NLR, LMR, PLR, SII, which are derived from them. The NLR, LMR, PLR and SII were calculated using the following formulas: NLR = ANC/ALC, LMR = ALC/AMC, PLR = APC/ALC, SII = APC× ANC/ALC. These immune-related IBs were calculated for three time periods: approximately 1 month before RT or from the start of immunotherapy to the start of RT (pre-IBs), during RT (medio-IBs), and within 2 months after RT or from the end of RT to the beginning of the next line of treatment (post-IBs). We recorded IBs more than once in each period and then averaged them. The delta-IBs were calculated as (medio-IBs - pre-IBs) ÷ pre-IBs.

### 2.4 Response evaluation

Therapeutic responses were evaluated based on RECIST 1.1. The physician’s follow-up included clinical assessments, enhanced computed tomography (CT) scans, esophageal barium meal, and other examinations, as needed. Additional imaging, including brain magnetic resonance imaging (MRI) and whole-body bone scan, was obtained based on symptoms, and the tumor responses were evaluated as complete response (CR), partial response (PR), stable disease (SD), or progressive disease (PD). PR and CR represented responsiveness, while SD and PD were defined as non-responsive. Short-term efficacy was defined as responses within three months after RT or until the beginning of the next line therapy. We used disease control rate (DCR), including SD, PR, and CR, to represent the maximum responsive population.

### 2.5 Statistical analysis

All statistical analyses were performed using IBM SPSS Statistical software, version 25.0. The correlation between the different irradiated groups and IBs was analyzed using chi-square test. The difference among irradiated groups was identified by pairwise comparisons. The cutoff values for delta-NLR, delta-LMR, delta-PLR, and delta-SII were defined using the receiver operating characteristic (ROC) curve. Univariate and multivariate logistic regression analyses were used to evaluate the associations between variables and short-term efficacy. Variables with a p-value < 0.05 in the univariate analysis were included in the multivariate analysis using backward stepwise model selection. Odds ratios (ORs) were reported with 95% confidence intervals (CI). Significance was defined as a p-value of 0.05 or lower.

## 3 Results

### 3.1 Patient characteristics

In total, 121 patients with advanced ESCC who met the inclusion criteria were enrolled in this retrospective study. Detailed clinical characteristics of the patients are presented in **Table 1**. The majority of patients were male (n=113, 93.4%), and the median age was 59 years. First-line immunotherapy was employed for 52 patients (43.0%). In all, 92.6% (n=112) of patients were diagnosed with stage IVB.

**Table 1:** Baseline demographics and clinical characteristics.

Characteristics	No. (%)
Sex	
Male	113 (93.4)
Female	8 (6.6)
Age (years)	
≤60	73 (60.3)
>60	48 (39.7)
Smoking status	
Never	46 (38.0)
Current or former	75 (62.0)
Drinking status	
Never	49 (40.5)
Current or former	72 (59.5)
Karnofsky score	
≥80	114 (94.2)
<80	7 (5.8)
Treatment mode	
Immunotherapy prior to RT	97 (80.2)
RT concurrent with Immunotherapy	24 (19.8)
Treatment line of immunotherapy	
First-line	52 (43.0)
Second-line and more	69 (57.0)
Stage of disease	
IVA	9 (7.4)
IVB (M1)	112 (92.6)
Total RT dose (Gy)	
Dose <30 Gy	2 (1.7)
30 ≤ dose <50 Gy	31 (25.6)
Dose ≥50 Gy	88 (72.7)
Irradiated sites	
Drainage area lymph node (esophagus)	33 (27.3)
Non-area lymph node (esophagus)	17 (14.1)
Drainage area lymph node and non-area	24 (19.8)
lymph node (esophagus)	
Bone	17 (14.1)
Brain	10 (8.3)
Liver	9 (7.4)
Lung	6 (4.9)
Soft tissue	5 (4.1)

RT, radiotherapy.

### 3.2 Irradiated site correlation with IBs

To analyze the relationship between irradiated organs and IBs, the patients were divided into eight groups based on the irradiated sites: drainage area lymph node (esophagus), non-area lymph node (esophagus), drainage area lymph node and non-area lymph node (esophagus), bone, brain, liver, lung, and soft tissue. IBs were converted into binary variables according to the medians. Chi-square test was used to analyze the correlation between IBs and RT groups (**Table 2**). Three of the IBs were related to the irradiated sites, the p-value of delta-LMR, delta-SII, and delta-ALC was 0.009, <0.001, and 0.018, respectively.

**Table 2:** Correlation between IBs and RT groups.

Characteristics	delta-NLR	delta-LMR	delta-PLR	delta-SII	delta-AEC	delta-WBC	delta-ALC	delta-ANC	delta-AMC	delta-APC
X^2^	15.506	18.599	5.252	21.353	5.246	11.756	16.368	10.175	6.725	8.501
p- value	0.072	**0.009**	0.649	**<0.001**	0.591	0.090	**0.018**	0.126	0.414	0.324

The p-value means inflammatory biomarkers differ in specific irradiated sites via chi-square test.

IB, inflammatory biomarker; RT, radiotherapy; NLR, neutrophil-to-lymphocyte ratio; LMR, lymphocyte-to-monocyte ratio; PLR, platelet-to-lymphocyte ratio; SII, systemic immune inflammation index; AEC, absolute eosinophil count; WBC, white blood cell count; ALC, absolute lymphocyte count; ANC, absolute neutrophil count; AMC, absolute monocyte count; APC, absolute platelet count. Bold: p-value<0.05.

**Figure 1:**
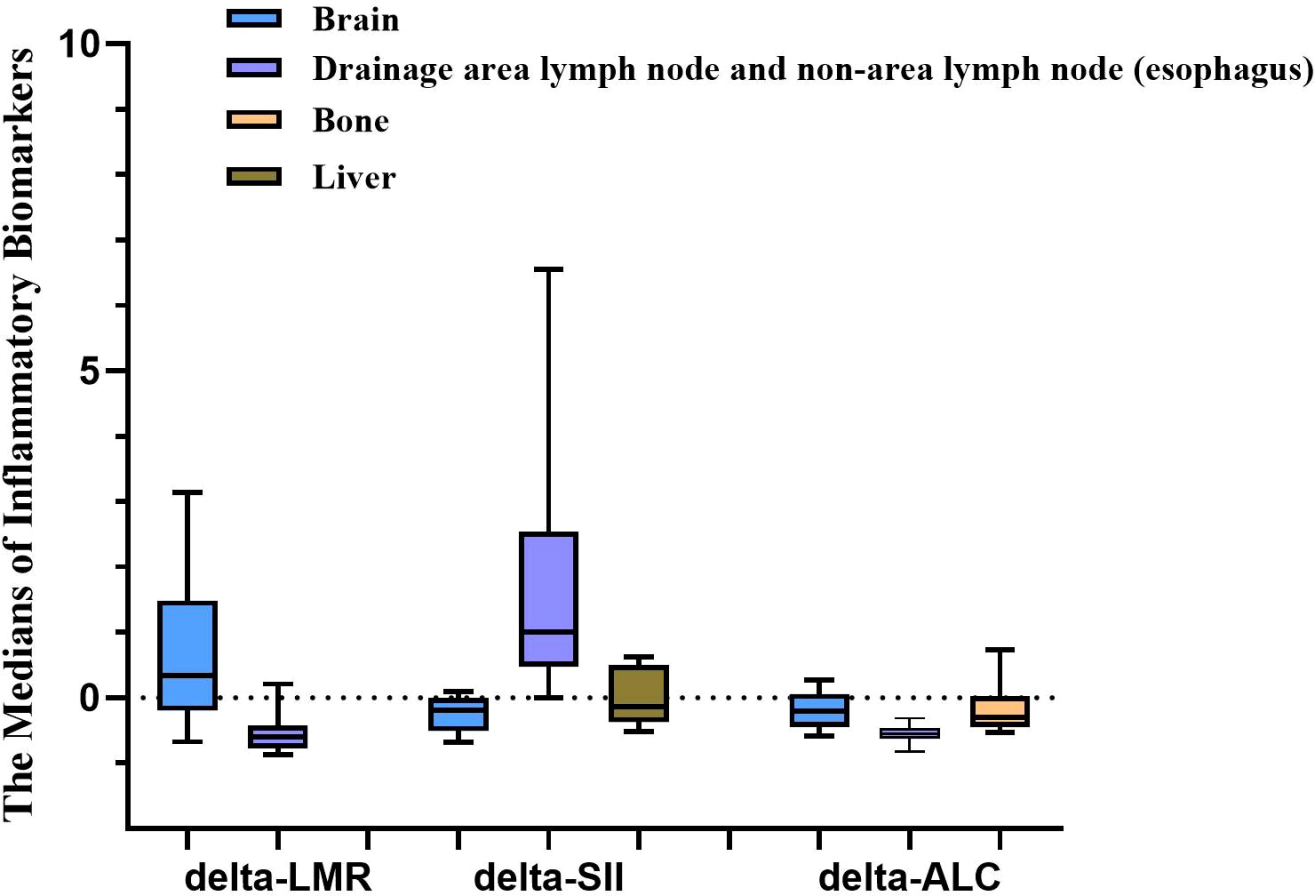
Statistical differences among irradiation sites existing in three inflammatory biomarkers (IBs). The medians of the delta-LMR, and delta-ALC in the brain irradiation group were the highest compared with the other groups, while it was the lowest in delta-SII. LMR, lymphocyte-to-monocyte ratio; SII, systemic immune inflammation index; ALC, absolute lymphocyte count.

Pairwise comparisons were performed within groups based on the above three indicators. As shown in **Figure 1**, there were statistical differences between the brain irradiation group, and the drainage area lymph node and non-area lymph node (esophagus) group. The brain irradiation group showed the highest medians of delta-LMR, and delta-ALC and the lowest median of delta-SII when compared to the other groups.

### 3.3 IBs correlation with therapeutic response

**Figure 2:**
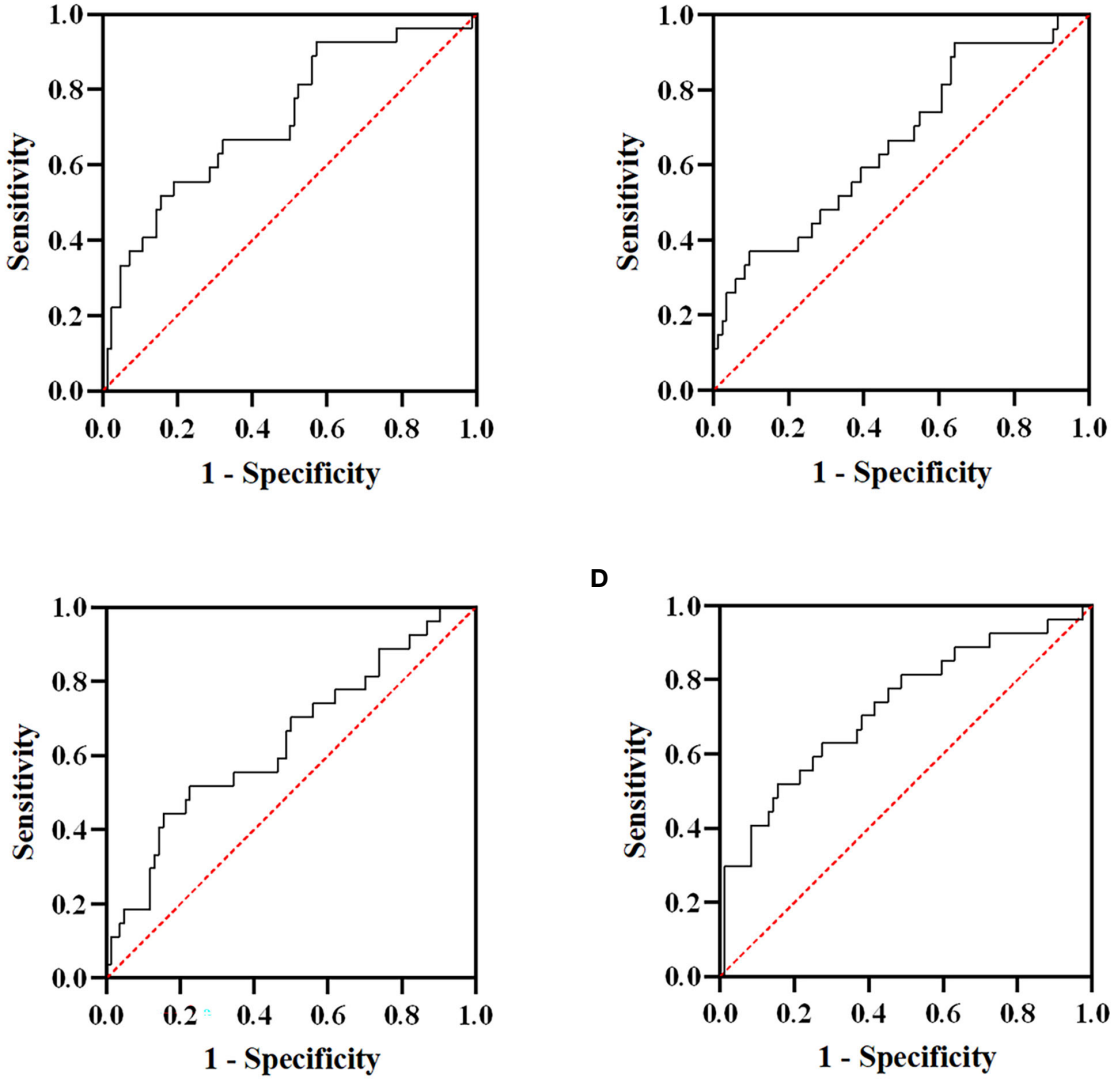
Receiver operating characteristic (ROC) curves of delta-NLR **(A)**, delta-LMR **(B)**, delta-PLR **(C)**, and delta-SII **(D)** for short-term efficacy. The areas under the curve (AUCs) for delta-NLR, delta-LMR, delta-PLR, and delta-SII were 0.723, 0.661, 0.640, and 0.725, respectively. NLR, neutrophil-to-lymphocyte ratio; LMR, lymphocyte-to-monocyte ratio; PLR, platelet-to-lymphocyte ratio; SII, systemic immune inflammation index.

**Figure 3:**
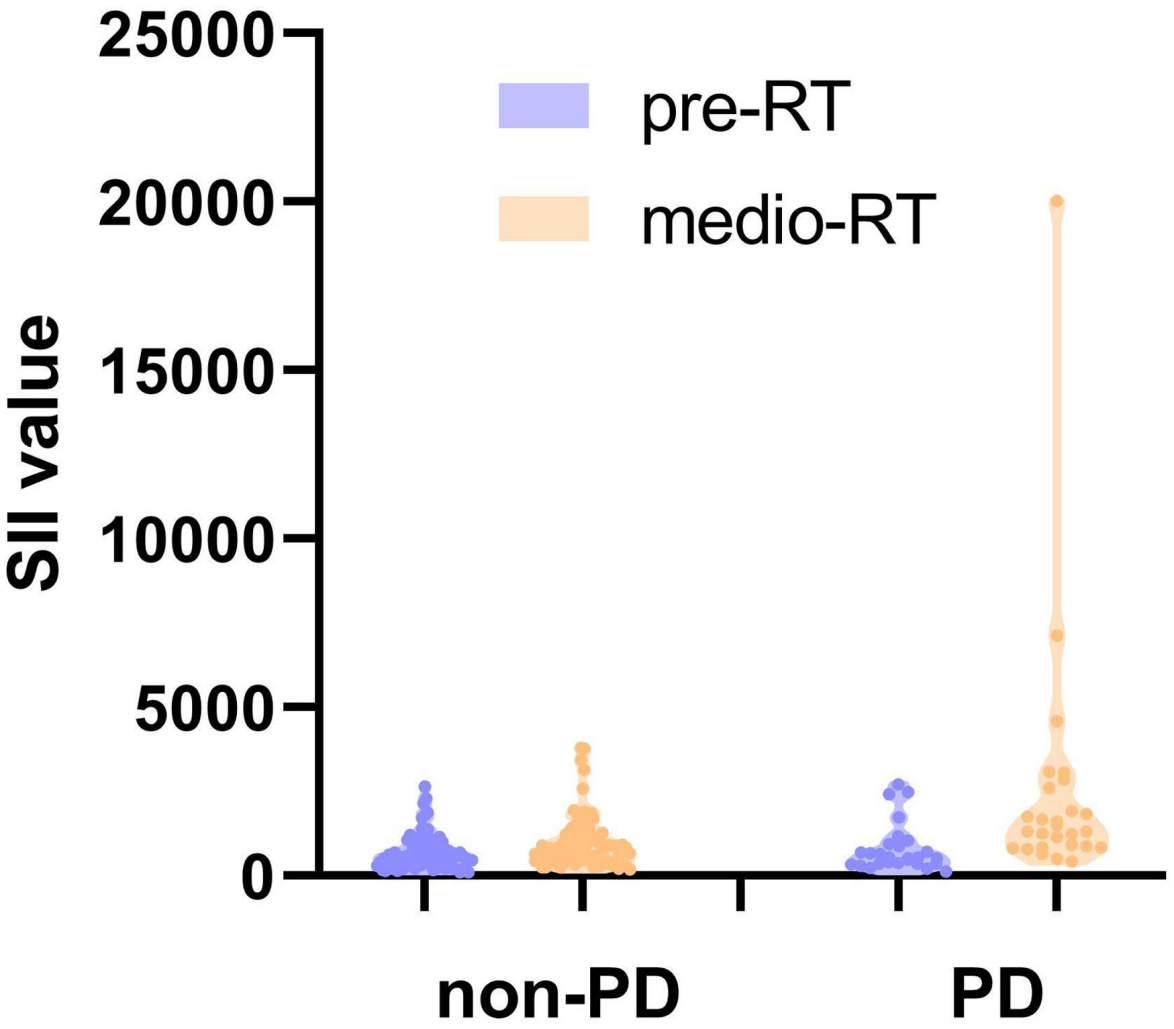
Changes of the SII value during treatment in non-PD and PD group. SII, systemic immune inflammation index; RT, radiotherapy; PD, progressive disease.

Of the 121 advanced ESCC patients, only one reached CR, 20 patients achieved PR, 70 patients showed SD, and 30 patients developed PD, yielding an overall response rate (ORR) of 17.4% and a DCR of 75.2%. The ROC curve was used to analyze the short-term efficacy of delta-IBs. The areas under the ROC curve (AUCs) for delta-NLR, delta-LMR, delta-PLR, and delta-SII were 0.723 (95% CI, 0.609–0.836; p = 0.001), 0.661 (95% CI, 0.542–0.781; p = 0.012), 0.640 (95% CI, 0.516–0.764; p = 0.029), and 0.725 (95% CI, 0.608–0.841; p < 0.001), respectively (**Figure 2**). The AUC value <0.7 was considered to indicate inferior performance for model prediction. Herein, we reserved the delta-NLR and delta-SII to assess their predictive value for short-term efficacy. In univariate analysis, the treatment line of immunotherapy (OR, 4.089; 95% CI, 1.528-10.943; p = 0.005), delta-NLR (OR, 4.244; 95% CI, 1.685-10.690; p = 0.002), and delta-SII (OR, 5.882; 95% CI, 2.255-15.344; p < 0.001) were significantly associated with short-term efficacy (**Table 3**). No significant differences were found between the irradiated sites and short-term efficacy. In multivariate analysis, the treatment line of immunotherapy (OR, 4.852; 95% CI, 1.595-14.759; p = 0.005) and delta-SII (OR, 5.252; 95% CI, 1.048-26.320; p = 0.044) were correlated with short-term efficacy. We further observed changes in the SII value during treatment, and an increase in the SII value was observed in the PD group (p < 0.001), indicating poor efficacy (**Figure 3**).

**Table 3:** Uni- and multivariate logistic regression analysis of clinical characteristics and inflammatory parameters with short-term efficacy.

Parameters	univariate analysis		multivariate analysis	
	OR (95%CI)	p-value	OR (95%CI)	p-value
Sex				
** female**				
** male**	2.417 (0.285-20.487)	0.418		
Age (years)				
** >60**				
** ≤60**	0.697 (0.293-1.658)	0.415		
Smoking status				
** never**				
** current or former**	1.972 (0.794-4.899)	0.144		
Drinking status				
** never**				
** current or former**	1.028 (0.443-2.385)	0.949		
Karnofsky score				
** <80**				
** ≥80**	1.229 (0.226-6.687)	0.812		
Treatment mode				
** Immunotherapy prior to RT**				
** RT concurrent with Immunotherapy**	0.811 (0.273-2.412)	0.707		
Treatment line of immunotherapy				
** First-line**				
** Second-line and more**	4.089 (1.528-10.943)	**0.005**	4.852 (1.595-14.759)	**0.005**
Stage of disease				
** IVA**				
** IVB (M1)**	1.167 (0.229-5.946)	0.853		
Irradiated sites				
** Drainage area lymph node (esophagus)**		0.652		
** Non-area lymph node (esophagus)**	0.889 (0.084-9.444)	0.922		
** Drainage area lymph node and non-area lymph node (esophagus)**	1.231 (0.105-14.424)	0.869		
** Bone**	2.400 (0.231-24.964)	0.464		
** Brain**	2.182 (0.197-24.208)	0.525		
** Liver**	0.444 (0.022-9.032)	0.598		
** Lung**	1.143 (0.077-16.947)	0.923		
** Soft tissue**	0.800 (0.037-17.196)	0.887		
** delta-NLR**	4.244 (1.685-10.690)	**0.002**	1.474 (0.304-7.152)	0.630
** delta-SII**	5.882 (2.255-15.344)	<**0.001**	5.252 (1.048-26.320)	**0.044**

OR, odds ratio; CI, confidence interval; NLR, neutrophil-to-lymphocyte ratio; SII, systemic immune inflammation index. Bold: p-value<0.05.

## 4 Discussion

As hematological IBs can reflect changes in the immune system, we used delta-IBs, including delta-NLR, delta-LMR, delta-PLR, delta-SII, delta-AEC, delta-WBC, delta-ALC, delta-ANC, delta-AMC, and delta-APC, to examine the immune activation of RT at different sites and their predictive effect on short-term efficacy. We found that brain irradiation may stimulate stronger immune activation than other extracranial organs, and lower delta-SII and earlier lines of ICIs were found to be independently associated with better short-term efficacy in patients with advanced ESCC who received RT and immunotherapy. To the best of our knowledge, this retrospective study is the first to investigate the immune activation of different irradiated sites as well as the relationship between delta-IBs and short-term efficacy in patients with advanced ESCC receiving radioimmunotherapy, and it may provide useful instructions for implementing individualized treatment regimens.

The current normative treatment for advanced ESCC is a combination of PD-1 agents and platinum-based chemotherapy agents. RT has historically been used to relieve the symptoms of relapse and metastases. Evidence suggests that radiation may eliminate tumors by activating local and/or systemic immune responses, particularly when combined with immunostimulatory agents such as ICIs. Many preclinical studies have shown that radiation may modulate the tumor microenvironment by enhancing the release of neoantigens, upregulating the expression of MHC molecules in cancer cells, increasing effector T-cell infiltration, and activating the cyclic GMP-AMP synthase (cGAS) and stimulator of interferon genes (STING) innate immune response (28). Consistent with our study, Wu et al. showed that brain irradiation induced the best immune activation effect when compared to other organs in advanced NSCLC (12). For strong immune activation of the brain in advanced ESCC, we speculated that irradiation of the brain destroyed the blood-brain barrier (BBB); thus, ICIs were able penetrate the brain and exert their pharmacodynamic effect (29). Therefore, patients with advanced ESCC and brain metastasis, even without symptoms, should receive RT as early as possible to activate the immune system. A clinical trial with a larger sample size is warranted.

Elevated SII denotes increased neutrophil and platelet counts and/or lymphocytopenia. Neutrophils have been shown to accelerate tumorigenesis by releasing genotoxic DNA substances, stimulating tumor cell proliferation by secreting PGE2, activating tumor angiogenesis by releasing Bv8 and matrix metalloproteinase 9 (MMP9), and promoting tumor cell migration, invasion, and extracellular matrix degradation (30). Tumor-activated platelets contribute greatly to tumor progression, metastasis, and immunosuppression via C-type lectin-like immune receptor 2 (CLEC- 2) (31). Contrary to neutrophils and platelets, T-lymphocytes have been shown to inhibit tumor proliferation and metastasis, induce cytotoxic cell death, and foster antitumor immune responses (32). In recent years, a growing number of studies have demonstrated the predictive value of SII in patients with ESCC undergoing surgery and neoadjuvant therapy (33–36). However, limited studies are available that use delta-SII to predict the efficacy for any tumor. In locally advanced non-squamous NSCLC, Biswas et al. observed that SII is an informative mid-treatment marker of overall survival and progression-free survival (37). Wang et al. found that the pre-/post-RT SII ratio and mid-RT SII ratio were potentially effective markers for predicting ESCC prognosis (33). Similarly, in this study, an elevated delta-SII, referring to the changes in SII before and during RT, indicated worse short-term efficacy in patients with advanced ESCC who received radioimmunotherapy. The AUC of the delta-SII was maximal in these four indicators, suggesting delta-SII has the best predictive value for short-term efficacy in this population.

Of the enrolled 121 patients, six received SBRT with a DCR of 83.8%, including four SD and one PR patients; five of them received HFRT with a DCR of 80.0%, including three SD and one PR patients, while the DCR in patients who received CFRT was 74.5%. Better efficacy was observed in patients who underwent SBRT and HFRT. Limited by the small sample size, more convincing research with a larger population is necessary to clarify the currently unclear mechanism. Regrettably, we did not find any significant association between short-term efficacy and different irradiation sites in this study. A possible explanation might be that metastases of EC mainly involve lymph nodes, while bone, liver, and brain metastases are relatively few.

Our study had several limitations. First, because of its retrospective nature, it was inevitably affected by loss of data, clinical bias of treatment choice, and unavailability of fresh serum specimens, which leads to a lack of molecular research. Moreover, the follow-up of our study was not long enough, as this study mainly explored the relationships between characteristics and short-term efficacy, not survival. Third, chemotherapy regimens were not recorded in this study, and different systemic regimens among the irradiation groups may have affected therapeutic efficacy. Therefore, a prospective study with a large sample size, more detailed RT parameters, such as dose, fraction, and sequencing of radiation combined with immunotherapy, and more molecular-biological index is needed to investigate the mechanism and optimal combination therapy strategy in advanced ESCC.

In conclusion, for patients with advanced ESCC receiving radioimmunotherapy, brain irradiation may trigger stronger immune activation than that of extracranial organs. Delta-SII and line of ICIs have predictive value for short-term efficacy, which may provide guidance for individualized treatment regimens.

## Data availability statement

The raw data supporting the conclusions of this article will be made available by the authors, without undue reservation.

## Ethics statement

The studies involving human participants were reviewed and approved by Institutional review board of Shandong Cancer Hospital and Institute. Written informed consent for participation was not required for this study in accordance with the national legislation and the institutional requirements.

## Author contributions

XM and XH designed the study and provided funding support. ML, GC and ZG collected and analyzed the data of the clinical trial. ML drafted the manuscript. XM and XH reviewed and edited the manuscript. All authors contributed to the article and approved the submitted version.
